# Social Cognition and Schizophrenia: Unresolved Issues and New Challenges in a Maturing Field of Research

**DOI:** 10.1093/schbul/sbaa034

**Published:** 2020-03-05

**Authors:** Anja Vaskinn, William P Horan

**Affiliations:** 1 Norwegian Centre for Mental Disorders Research, Oslo University Hospital, Oslo, Norway; 2 Institute of Clinical Medicine, University of Oslo, Oslo, Norway; 3 VeraSci Inc, Durham, NC; 4 Department of Psychiatry and Biobehavioral Sciences, University of California Los Angeles, Los Angeles, CA

**Keywords:** schizophrenia, neuropsychology, cognition, social functioning

## Abstract

Social cognition has become a topic of widespread interest in experimental and treatment research in schizophrenia over the past 15 years. This explosion of interest largely reflects the robust evidence that social cognition is among the strongest known correlates of poor community functioning throughout the course of schizophrenia. While progress has been impressive, we consider several fundamental questions about the scope, structure, and optimal measurement of social cognition that remain unanswered and point to the need for continued method development. We also consider more recently emerging questions about individual differences, ecological and cross-cultural validity, and intervention approaches, as well as broader technological changes that impact how we understand and use social cognition at a societal level. Continued efforts to creatively grapple with the complexities and challenges the field now faces hold great promise for helping us understand and more effectively treat a major source of functional disability in schizophrenia.

## Introduction

Social cognition as a topic of interest in schizophrenia research gained momentum after two National Institute of Mental Health (NIMH)-sponsored workshops in the mid-2000s.^1,2^ Both workshops recommended agendas for future research. An early short-term goal was to achieve agreement on definitions and domains of social cognition,^1^ accomplished with the second workshop.^2^ Other proposed research goals included investigation of psychometrics and the refinement of existing measures, describing the social cognitive impairments in schizophrenia (timing, associations with symptoms and functioning, and comparisons with other mental disorders), examining the factor structure of social cognition, translational studies to identify neural substrates, and treatment development.^1,2^

Since then, the field has witnessed an enormous rise in scientific interest. Entering the search term “social cognition AND schizo*” in PubMed gives 38 hits for the year 2000 and 341 for the year 2018. This *In Translation* article briefly takes stock of the impressive accomplishments of social cognitive research in schizophrenia over the past 15 years, describes some key issues and challenges that this maturing field currently faces, and looks forward to the next generation of research in this vibrant area.

## What We Know

Over the past 15 years, our understanding of social cognitive impairments in schizophrenia, including their magnitude and course, functional significance, and amenability to intervention, has dramatically expanded in several ways. First, it is now clear that people with schizophrenia demonstrate substantial impairments in three areas of social cognition defined by the NIMH workgroup.^2^ Dozens of studies show large impairments on performance measures of emotion perception/processing (*d* = 0.89) and mentalizing (*d* = 0.96), while a much smaller number of studies show a large impairment in social perception (*n* = 12; *d* = 1.04). A fourth area, attributional style, was also recognized by the workgroup but, as discussed below, impairment is not consistently found in this area.^3^ Neuroimaging studies show corresponding patterns of abnormal regional activation and connectivity during the performance of these social cognitive tasks in psychosis.^4^ More recent studies suggest that the impairments extend to many other social cognitive domains, such as empathic accuracy and self-referential processing.^5,6^

Second, converging evidence indicates that emotion perception/processing, mentalizing, and social perception impairments are trait-like features of schizophrenia. The impairments seen in chronically ill individuals are present at similar levels during the early postonset phase, are longitudinally stable, are not secondary to factors such as medication effects, and are detectable at attenuated levels in unaffected biological relatives.^7^ There is growing evidence that social cognitive impairments are present in prodromal individuals at heightened risk for psychosis and are possible predictors of conversion to psychosis.^8,9^

Third, social cognition is a key correlate and predictor of functional outcome. Similar to the well-established link between nonsocial cognitive deficits and functioning, social cognitive impairments consistently relate to how well-affected individuals function in their daily lives both cross-sectionally and longitudinally.^10,11^ In fact, a meta-analysis reported that social cognition shows an even stronger link to functioning, accounting for about 16% of the variance in community functioning compared to 6% for nonsocial cognition.^12^ Along these lines, there is now compelling evidence that social cognition mediates the relationship between nonsocial cognition and functional outcome, with approximately 25% of the variance in the functional outcome being explained by such multifactorial models.^13^

Fourth, social cognitive impairments detected on performance measures are amenable to treatment. A recent meta-analysis of 16 studies using social cognitive training programs^14^ reported medium-to-large improvements in the two most commonly assessed domains, facial affect identification (*d* = 0.84 in 12 studies) and mentalizing (*d* = 0.70 in 13 studies). Notably, the social cognitive treatment-related gains are not accompanied by improvements in nonsocial cognition^14^ and, alternatively, nonsocial cognitive remediation does not result in significant social cognitive improvements.^15^ These findings converge to suggest that social and nonsocial cognition are distinct treatment targets that require different types of interventions. Beyond improvements in social cognitive task performance, preliminary evidence indicates that these interventions produce detectable structural and functional brain changes.^16^

## Current Challenges

Despite the rapid expansion of social cognition research in schizophrenia, some of the basic issues raised in the NIMH workgroups 15 years ago remain unresolved. Additionally, as in any maturing area of research, new issues and complexities have emerged. We focus on four unresolved basic issues and four more recently emerging questions.

### Basic Issues

One unresolved basic issue concerns the scope and profile of social cognitive impairments within schizophrenia. Although we highlighted several areas of impairment, not all aspects of social cognition appear to be markedly impacted. For example, affective empathy, ie, relatively automatic experience sharing or “simulation” processes through which observed actions and social/emotional cues trigger a shared neural response in the observer,^17–19^ may be relatively intact. It has been proposed that the mirror neuron system is centrally involved in the affective component of empathy.^20,21^ A number of studies have found normal neural responses in schizophrenia across experience-sharing tasks that range from observing simple motor actions to more complex social-emotional behaviors.^22–26^ As another example, as a group, individuals with schizophrenia do not demonstrate consistent impairment in the area of attributional bias (particularly hostility bias),^3,27^ one of the main areas defined in the original NIMH workshops. Pronounced attributional biases may be more specifically linked to experiencing paranoid beliefs.

Second, it remains unclear if there is a distinctive social cognitive profile in schizophrenia compared to other neuropsychiatric conditions. It is now well documented that social cognitive impairments are present across dozens of psychiatric, neurological, and developmental,^28^ as well as genetic disorders.^29^ Direct comparisons between schizophrenia and other disorders thus far provide a mixed picture. For example, although impairments consistently appear to be more severe in schizophrenia than bipolar disorder, the magnitude of the group differences are modest.^30^ Further, meta-analytic evidence indicates that schizophrenia and autism do not differ for mentalizing but that autism has larger impairments for emotion perception.^31^ However, recent direct comparisons using validated measures found similar social cognitive impairments across these disorders,^32^ though the similar performance deficits may be associated with different neural mechanisms^33^ and/or error types, such as overmentalizing or undermentalizing.^34^

Third, the basic structure of social cognition in schizophrenia remains unsettled. Factor analytic studies of social cognitive measures have been plagued by a lack of replicability, yielding one, two, three, or even four factors.^35^ It remains unclear to what extent the number of factors identified reflects methodological issues (eg, the specific tasks included, the frequent use of a narrow range of social cognitive tasks, and variability in sample characteristics) vs the actual structure of social cognition. Most larger studies using measures from multiple social cognitive domains report multifactorial, rather than unitary, solutions but more large-scale studies that include measures from all of the theoretically proposed domains are needed to resolve this structural issue.

Fourth, most available social cognitive measures have substantial limitations for use in schizophrenia. Many commonly used measures were borrowed from other fields (eg, childhood neuropsychiatric disorders) and their validity and psychometric properties (eg, floor/ceiling effects and test–retest reliability) in schizophrenia are often inadequate or simply unknown. An impressive effort to address this measurement issue was the Social Cognition Psychometric Evaluation (SCOPE) study,^36,37^ which identified existing tests worthy of comprehensive evaluation, either “as is” or with modifications, for potential use as clinical trial endpoints. Of eight candidate measures, only two emotion processing tasks and one mentalizing task showed acceptable properties for clinical trial use though; even these measures are not without limitations. Further development was recommended for other tasks. Another initiative, the Social Cognition and Functioning in Schizophrenia (SCAF) study,^38^ sought to create new measures by adapting paradigms from social neuroscience for use in schizophrenia clinical trials. Of the four adapted paradigms examined, only one, an empathic accuracy task, showed acceptable properties,^39,40^ while the others require further development.

Overall, these unresolved basic questions have important practical consequences. The suboptimal test characteristics and unknown factor structure make it difficult to provide guidance for researchers or clinicians interested in selecting a battery of tests to adequately measure “social cognition” in schizophrenia. The paucity of measures with adequate properties for clinical trial use also hinders efforts to convincingly demonstrate the efficacy of new interventions. Although the SCOPE and SCAF initiatives demonstrate the substantial challenge of establishing measures that are suitable for use in schizophrenia, the development of new measures is a pressing need.

### New Challenges

As the research base has rapidly accumulated over the past 15 years, several new challenges have arisen. Further, there have been major technological advances throughout the world that directly impact how we communicate with each other and use social cognition in daily life. Looking forward to the next 15 years of research, we conclude by highlighting four questions that have more recently emerged.

#### How Should We Deal With the Heterogeneity of Social Cognition in Psychosis?

It has become clear that there is considerable variability in social cognitive task performance among those with schizophrenia. One approach to addressing heterogeneity focuses on identifying subgroups with distinct profiles within schizophrenia.^41,42^ For example, some studies have reported on a subgroup seemingly without social cognitive impairments.^43,44^ Notably, in the area of nonsocial cognition, schizophrenia researchers have similarly debated for years about the possible existence of a “neuropsychologically normal” subgroup. At this stage, a number of studies using various designs suggest that virtually all individuals with schizophrenia, including those with superior intelligence,^45^ experience some degree of cognitive deterioration. For example, 98% of people with schizophrenia fail to reach the nonsocial cognitive level expected from estimated premorbid intellectual function or maternal education.^46^ This issue is much more challenging to address for social cognition because we do not have well-established indices of expected social cognitive ability, and the clinical value of subgrouping remains to be seen. In light of the considerable variability in social cognition among those with schizophrenia, it would be desirable to develop assessment batteries that can identify profiles of both weaknesses and strengths at the individual level. Such batteries could guide interventions that capitalize on an individual’s relative social cognitive strengths.

Another approach to heterogeneity focuses on conceptualizing social cognitive processes as broad transdiagnostic constructs. The NIHM workgroups occurred in a largely pre-Research Domain Criteria (RDoC) world. The RDoC initiative was intended to inspire researchers to look beyond conventional diagnostic categories and, instead, focus on integrating behavioral and biological components of basic dimensional constructs that span the full range of normal to abnormal functioning. Several social cognitive constructs are included within the social processes domain of the RDoC matrix.^47^ At this stage, only a few studies have examined social cognition from an RDoC perspective across the psychosis spectrum. While early results, primarily in the area of facial emotion processing^48–50^ show promise, the value of this approach remains to be determined.

#### How Should We Address the Ecological and Cross-Cultural Validity of Social Cognitive Measures?

Linking performance on social cognition tasks not to biology, but to real-life, is at the core of ecological validity. Despite the dynamic, interactive, and rapidly fluctuating nature of social cognition in daily life, most commonly used measures in schizophrenia, including those endorsed in the SCOPE project, involve viewing and responding to static stimuli (eg, labeling emotions in faces or eyes) or interpreting written social vignettes (eg, making inferences about a person’s true intention). These types of tasks have been criticized in schizophrenia research and beyond for their limited ability to capture social cognitive processes as they typically unfold in daily life.^51,52^ In addition to developing laboratory tasks using more representative dynamic stimuli,^53^ alternative methods, such as experience sampling and virtual reality are emerging in an effort to enhance ecological validity.^54^ Further, the global availability of smartphones opens up the possibility of new digital phenotyping metrics (eg, calls/texting and social media use) of social cognition and behavior.^55,56^

A related concern is the cross-cultural validity of social cognitive tasks. Research has been dominated by work in Western cultures, especially the United States, and remarkably few studies have conducted direct cross-cultural comparisons. Although numerous social cognitive tasks have been translated for use in other countries, it is quite possible that they measure somewhat different constructs when administered in different languages or contexts with different social norms. Several studies have found an impact of race, culture, and social class on social cognition.^57–60^ It is critical that we carefully attend to potential cultural biases when interpreting task scores and developing new measures.^61^

#### How Can We Develop More Effective Social Cognitive Interventions?

As treatment research has matured, early enthusiasm about training programs to improve social cognition and community functioning has been tempered. For example, a closer look at training interventions for social cognition reveals substantial methodological limitations (eg, frequent use of small sample sizes, nonexperimental designs, and nonactive control conditions^62^). Further, recent studies using larger samples and more rigorous designs have often shown notably weaker effects on social cognitive task performance and there is currently not much compelling evidence of generalization to meaningful improvements in functioning.^63,64^ Thus, the complexities of improving targets as complex as social cognition have come to the fore. To enhance the impact and generalizability of social cognitive training, initial creative efforts have attempted to incorporate new technologies (eg, virtual-reality-based exercises^65,66^) and implement novel bridging activities conducted outside of the clinic. Fully computerized interventions that can be independently completed using portable electronic devices have also been developed. While questions may be raised about the viability of these approaches—eg, can interacting with a computer training program truly generalize to improvements in real-world social interactions?—initial results have been encouraging.^67,68^

The picture has been similar for pharmacological treatment approaches, which has been dominated by a focus on oxytocin. Following a proliferation of single and repeated dose studies that built on early excitement about the benefits of oxytocin, enthusiasm has been tempered considerably by research literature in schizophrenia that is now decidedly mixed.^69,70^ Again, the complexities of oxytocin have come to fore, with current research focused on more fine-grained questions, such as identifying optimal social cognitive treatment targets, dosing, and administration parameters (eg, in combination with psychosocial treatment).^71,72^ Looking forward, we also anticipate an expansion of interest beyond oxytocin to other molecules as basic research into the pharmacology of social cognition continues to grow.^73–77^ There is also emerging interest in brain stimulation approaches to enhancing social cognition in schizophrenia, though findings thus far have been mixed.^78–80^

#### How Should We Investigate Social Cognition in an Increasingly Digital Society?

Human communication has changed markedly since the mid-2000s. Personal smartphones are now extremely common across the globe and an increasingly large quantity of our social interactions occur online and through various forms of social media. As a consequence, the means through which we communicate and use our social cognitive abilities are dramatically shifting. As noted above, these technological advances open up exciting new vistas for assessing social cognition through digital phenotyping or delivering digital interventions.

In line with our increasingly computer-mediated interpersonal communication and relationships, the social cognitive skills that are most important in our daily lives are also rapidly shifting.^81–83^ For example, when we post on Facebook or Instagram, we consider how others at widely varying levels of closeness to us will react. When interpreting others’ intentions and beliefs from brief posts, emojis, or “likes”/absence of “likes,” we have far fewer social cues to draw upon than if we were directly interacting. We consider issues such as whether it is socially appropriate to use emojis or abbreviations (“tmi,” “btw,” and “lmk”) with, eg, a friend, an online friend, an acquaintance, or a supervisor. These new forms of communication can be just as important for adaptive functioning as face-to-face interactions, and the sociocultural norms that guide them are evolving. We anticipate an expansion of attention to digital social cognition in schizophrenia research in the next 15 years. This is particularly important for younger individuals, including those at risk for or with a relatively recent onset of psychosis because their generation has not experienced a world without digital technology and social media.
